# Study on the Influence of Government Intervention on the Occupational Health and Safety (OHS) Services of Small- and Medium-Sized Enterprises (SMEs)

**DOI:** 10.1155/2018/5014859

**Published:** 2018-10-25

**Authors:** Jingjing Zhang, Qiang Mei, Suxia Liu, Qiwei Wang

**Affiliations:** School of Management, Jiangsu University, Zhenjiang 212013, China

## Abstract

The OHS services of SMEs are still in their start-up stage in China. As such, there is an absence of mature market norms, which in turn makes it difficult to guarantee the quality of OHS services. The government, as the “night watchman” of the market, is supposed to not only involve itself in the regulation of OHS service quality, but also introduce and implement proper regulatory strategies. This paper employs a computational experiment approach to construct an experimental platform based on multiagent interactions. By simulating the OHS service transaction activities of SMEs, this paper takes the perspective of dynamic evolution. From this perspective, we probe into the optimal regulatory strategy covering the positive influence of government punishment, policy supports, and service quality ratings of the OHS services of SMEs. These strategies should be built on the foundation of proper punishment standard and intensity, proper support standard and intensity, and quality rating information disclosure.

## 1. Introduction

More and more Chinese companies actively adopt OHSAS18001 system in order to adjust to the international competition order. However, Chinese small- and medium-sized enterprises (SMEs) are confronted with various difficulties in building the occupational health and safety management systems (OHSAS) due to the limitations in scale, labor power, and economic power. At present, many countries' governments are trying to shift the corporations' occupational health and safety responsibilities to private agencies, so as to lessen the “legislative burden” and use the “market” to improve occupational health & safety (OHS) system [1]. Researches show that the marketized supply of OHS services helps the corporations prevent safety accidents, reduce cost, save time, facilitate the employees' flexibility, and improve the overall quality of OHS plan [2, 3]. Successful OHS services employ the service provider's unique expertise and resources to help the corporations actuate their occupation health and safety goals [4]. Thus, to help SMEs strengthen their international competitiveness, and also to turn around their historically poor safety records, the Chinese government encourages SMEs to seek professional OHS services from third-party service agencies. These agencies can help facilitate SMEs' construction, operation, and improvement of their OHSAS18001 OHS management systems. In other words, these third-party agencies can help SMEs provide a professional supply of OHS services. It is in this context that OHS services develop rapidly in China which has become an important means of improving SMEs' OHSAS18001 management systems.

However, China's OHS services are still in the preliminary stages. More time is needed for the industry norms and self-discipline of the OHS service market to take form. In addition, combined with the apparent lagging of OHS service effect and the high degree of information asymmetry related to service processes, there is a severe lack of any reliable means for purchasers to distinguish different SMEs' service quality. Due to the combination of these factors, guaranteeing the quality of OHS services is difficult. In addition, the risk SMEs face when purchasing low-quality OHS services is significant [5]. When low-quality OHS services are purchased by an SME, this not only makes it difficult to avoid safety risks, but also increases the SMEs' safety costs and dampens their enthusiasm for purchasing OHS services [6, 7]. The practice of purchasing OHS services also departs from the original intention of the Chinese government when they began encouraging the development of OHS services. Thus, there is an urgent need to reduce the risk of purchasing shoddy service, guarantee the service quality, and promote the well-being of OHS services market. The objectives of this study are how to address the above-mentioned issues.

In general, the quality control of products is uniformly conducted by the principal, as agreed in the contract. However, due to the complexity of safety-related accidents and the lagging of service effect, it is very difficult for SMEs (as the principal) to distinguish rights and liabilities and control service quality via commercial contracts, as would normally be the case with ordinary products. In this case, third-party intervention is needed to assist management. The quality of OHS services is a matter of concern as regards social public security. OHS service quality constitutes a branch of public administration, and the government has a compelling obligation in terms of public service management [8]. As such, it is logical for the government's functional departments to intervene in the management of OHS service quality. In consideration of the government's position in the OHS service market and the market response combined, which is different from the government's direct supervision of the corporations, it is an urgent theoretical and practical problem to explore what intervention measures the government should take to realize the virtuous development of OHS service market to help SMEs build the OHSAS18001 system efficiently. Therefore, this paper attempts to compare the intervention measures taken by the government in the development of the OHS service market and discuss which intervention measures can bring about effective functioning of the OHS service market system. Here, effective means that SMEs are willing to purchase the high-quality OHS service. In other words, SMEs are willing and able to establish the OHSAS18001 system efficiently through market mechanisms. Accordingly, in order to measure the effectiveness of different intervention, one needs to analyze whether or not enterprises will purchase services under a specific government intervention and the quality of the purchased services.

The OHS service market in the real world is a large and complex system that requires the cooperation of government, service organization, and SMEs. Due to the complexity of the system environment, the incompleteness of information among transaction agents, the behavioral bias and framing effect of experiential drive, and the nonlinear associations among elements, the evolution of the system presents an instability and polymorphic equilibrium. This means that both traditional empirical study method and game simulation are not applicable to the research on the effects of government intervention. In order to study the impact of government interventions from a dynamic and bounded rational perspective, this paper employed multiagent-based computational experiment simulation method. The purpose of this method is to construct a controlled and reproducible government intervention model for OHS service market based on heterogeneous subjects and simulate different interventions so that it is possible to observe the market status.

The contribution of this study is the tentative application of the multiagent-based computational experiment simulation method on the OHS service market studies. Through the simulated evolution based on real situations, it observes the market response when the government takes different measures, which further proves and compares which measure can genuinely promote the sound development of OHS service market, so as to help SMEs make their OHS plan efficiently. This study not only fills the research gap from a theoretical perspective but also informs policy-making and practice.

## 2. Literature Review

Through reviewing the literature, the researchers found that previous studies on OHS services and government regulation mainly focused on the supervision of OHSAS18001 certification system, which discussed the relationship between the government and the enterprises. Different from the direct supervision of the government on the enterprises, the government's participation in the OHS service market intervention is concerned with the triangular relationship between the government, enterprises, and OHS service organizations. Researches that are directly relevant to this study are few and far between. However, it should be noted that enterprise security resulting from enterprise OHSAS18001 certification is an important part of public security. Besides, OHS service market could also be considered as an important part of public service market. Therefore, the research on the OHSAS18001 certification system supervision and public service market supervision may serve to inspire the current study.

### 2.1. Studies on OHSAS18001 Certification System Supervision

The OHSAS18001 certification is an international standard jointly launched by 13 organizations, including the British Standard Institute (BSI) and the Det Norske Veritas (DNV). The purpose of this certification is to urge the enterprise that has received the certificates to ensure the maximum health security of its employees and adequately protect the property safety of itself.

A comparative study by Santos [9] focused on the participation of Portuguese small- and medium-sized companies in OHSAS18001 certification. The result shows that companies engaged in certification are better at preventing security risks than those that do not. Some researchers have studied how to motivate companies to participate in OHSAS certification. For example, Olsen [10] believes that the government plays an important role in guiding small businesses to participate in OHSAS certification. Kvorning [11] believes that small enterprises have limited resources and therefore are incapable of actively participating in OHSAS certification. However, government regulation, trade union intervention, and network assistance can encourage the initiative of the small enterprises. Several researchers have examined how to improve the level of OHSAS18001 certification. Hasle P [12] argues that the policy instrument which jointly combines government, business owners, and trade unions can effectively improve the certification level. Legg et al. [13] think that, in order to improve the certification level, it is necessary to consider the specific characteristics of SMEs and strengthen the government's inspection of the compliance of enterprises during the certification process. Okun [14] believes that government could participate in safety certification with institutes like trade organizations and trade union organizations and use the existing communication channels to address high-risk workplace safety and health issues which can improve a company's certification level. A few researchers have studied how to ensure the effectiveness of OHSAS certification. Some believe that the joint participation of government regulators and national security assessment departments, laws and regulations, policies, resources, information, organization, planning, and implementation are all key factors to guarantee the effectiveness of OHSAS certification [15–17]. When introducing government regulation, it is important to consider the diversity characteristics of SMEs [18]. Besides, the risk-oriented approach of government regulation focuses on regulatory standards development and enforcement activities on the highest priority risks through various risk-based indicators and policy tools, which makes regulation more proportional and effective [19]. Some researchers studied the failure of OHSAS certification system and found that if the policymakers are too optimistic about the operation of the compulsory certification, the regulation has its limitations, and the lack of institutional factors leads to operational failure [20–23]. In face of certification system failure, the government needs to strengthen the certification enterprise intervention. Not only should the regulation of the certification enterprise be strengthened, but the technical, financial, and material support to the certification enterprise should also be provided [24–27].

Previous studies have shown that the well-functioning of the OHSAS18001 certification system will effectively improve the safety level of SMEs, and the operation of the system needs to be strengthened by government regulation. However, whether it is to encourage enterprises to participate in certification or to prevent the certification system from failing through regulation, previous studies only focus on the relationship between the government and the enterprises, and the enterprises are regulated entities of the government. In the studies that further explore government regulation methods, the empirical methods or game methods are used to measure the effects of regulation under a single method. There is no comparison between different supervision methods, and no in-depth study on how to control the intensity and frequency of regulation strategies can be found.

### 2.2. Studies on Government Regulation of Public Service Market

Government purchase of public service is a new mode of providing services for citizens by the government, which solves the problem of the government being the only public services provider. The government's function has been changed greatly and it becomes more service-oriented due to the new mode [28]. However, it should be noted that the government purchase of public service does not mean that the government will transfer its responsibility to social organizations. Still it is supposed to shoulder the responsibility of fostering a competitive public service supply market, fully support public services, strengthen contract oversight, strengthen the capacity to manage throughout the procurement process, and improve the quality of services [29, 30], since the outsourcing of public service contracts faces various risks, such as moral hazard and rent-seeking that act against fair competition and equity risk [31]. Besides, the market is full of high uncertainties and challenges that may cause the deviation of government public service outsourcing. Therefore, the risk-based supervision of the government is needed to monitor enterprise performance and assess contractor performance [32, 33]. In the government-directed social supervision model, the government relies on administrative law enforcement to formulate market rules and standards, guides market credit and price mechanisms in the form of services and penalties, and makes public interests a starting point to disclose market information and induce rational production and consumption [32, 34, 35]. With regard to the supply of rural public goods in China, the grassroots government needs to better reflect its functions in establishing scientifically sound market approval and withdraw criteria, monitoring competition and price systems, and curbing corruption [36]. In the market-oriented operation of public sanitation services, the government fosters, regulates, develops, and supervises the market through price, financial subsidies, and taxation, in order to improve the quality of public sanitation services. In addition, the government also acts as a decision-maker of market rules, a procurement agent for public sanitation services, and a supervisor of market operation [37].

These studies often refer to the administrative and law enforcement means that is necessary for the government in different fields from various perspectives by means of case study, empirical research, and other methods. The government perform its market supervision duties through price guidance, information disclosure, financial subsidy incentives, and penalties for breach of laws and regulations. However, there is no in-depth study on the advantages and disadvantages of these regulatory methods and how to draw the lines for the supervision. Different from the traditional public service market, in the OHS service market, the government is not a direct service purchaser. The two sides of the market are SMEs and service organizations, respectively, and the purpose of government supervision is not only to protect the market, but also to encourage and help more SMEs to establish OHS18001 system certification efficiently. Therefore, OHS service market regulation faces more challenges than the general public service market regulation.

In summary, whether it is the OHSAS18001 certification system or public services, all researchers emphasize the importance of government regulation. However, few studies have analyzed the different intervention strategies that may guarantee the effective functioning of OHS services market from the perspective of government regulation. Different from direct supervision of the enterprise by the government, the priority at present is how to link the role of the government in the OHS service market with its market reaction and explore what kind of intervention strategy the government should adopt so as to achieve the sound development of the market, which are still theoretical and practical problems that need to be solved. Many current studies on the government supervision of OHSAS18001 certification system focus on the relationship between the government and enterprises by empirical or game analysis. The research on the supervision of public service market discusses the influence of government regulation from the perspectives of government punishment, government support, and market information disclosure. The environment considered is simple and static, which neither reproduces the dynamic and complex system situation nor compares the market operation effect under different intervention strategies.

In real life, as seen from the micro perspective, OHS service activities constitute a transaction subsystem that consists of two types of activities. Those two activities are the purchase of OHS services by SMEs and the provision of OHS services by third-party professional service agencies. As seen from the macro perspective, however, OHS service activities become a complex macro system that consists of numerous micro subsystems. Therefore, the traditional research methods are difficult to achieve the purpose of comparing intervention strategies and measuring the effect of market intervention. The multiagent-based computational experimental method adopted in this study is a method using computer technology to construct experiment object, experiment environment, and experiment platform, simulate the dynamic law of material movement in the real world, and carry out experimental research on scientific problems [38].

It is a research method in the natural sciences studies, mostly applied in the studies of uncertainty and network evolution. Now this method is gradually extending into the social sciences studies and is applied well in these fields, making it a useful tool in studying system complexity. For example, Wang and Singh [39] employed this method to expand the behavioral economics theory and yielded the financial mathematical model; Hafezi et al. [40] simulated the operation of financial market to predict the share price by constructing a four layer multiagent framework; Behdani et al. [41] used the multiagent-based modeling in the environmental management studies to depict the complexity of the government's management of environmental issues; Santos et al. [42] constructed multilayer multiagent models to study the change in behavior of different subjects in the electricity market; Meng et al. [43] developed multiagent-based modeling framework to simulate the operation of supply chains, comparing the competitive powers between supply chains; and Shafie-khah and Catalão [44] simulated the operation model of electricity market to compare the operation performances in different situations by depicting the behaviors of the service provider, supervisor, and relative participants based on complicated and changeful real situations. These studies all have made contribution to the application of this method in social sciences studies. Therefore, on the basis of existing research, this paper constructs a dynamic model of government participation in OHS service market supervision and selects three common intervention methods, i.e., punishment, government support, and market information disclosure, in order to test the market reaction under the three means. Not only can the advantages and disadvantages of various interventions be compared, but the strengths and limitations of various measures can also be better understood, not to mention the reference this research could provide for the government about the specific interventions. It is hoped that this study could promote the sound development of the OHS market and bring about the improvement in the quality of OHS enterprises.

## 3. Methods

### 3.1. Experimental Framework

**Figure 1:**
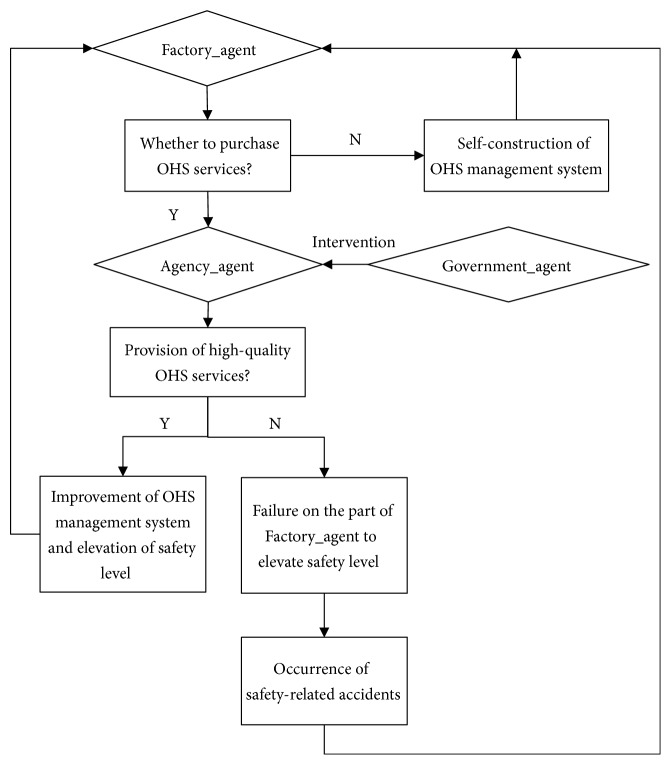
Interaction process of OHS service transactions.

This paper abstracts the transaction process of the OHS services of SMEs from reality for simulation purposes. The related agents involved therein include SMEs (Factory_agent), third-party professional OHS service agencies (Agency_agent), and the government (Government_agent). The interactive relationships between these agents are manifested as follows: (1) Agency_agent provides OHS services, and Factory_agent makes the decision whether or not to purchase OHS services. (2) When low-quality OHS services are purchased, Factory_agent faces safety-related accident risks. (3) Government_agent intervenes with regard to OHS services, and different intervening behaviors influence the OHS services of SMEs in different manners. See the specific interaction process in Figure 1.

In order to make the model closer to the real situation, the analysis is founded on the following five assumptions in the system development: (1) High-quality OHS services can effectively improve the OHSAS18001 safety system of SMEs and reduce the occurrence of safety-related accidents. (2) Due to requirements of relevant laws and safety system certification, there is no intentional reduction of OHS service quality after purchasing service of SMEs. (3) Due to capability limitations and information asymmetry, it is difficult for SMEs to independently distinguish the quality of the OHS services they have purchased. (4) In reality, when SMEs (due to limitations in terms of scale, manpower, and technology) choose to self-construct and implement their own OHSAS18001 safety systems, they have to pay a higher safety production cost. Therefore, it is assumed that their self-construction cost is higher than the cost would be to purchase OHS services. (5) The prices of OHS services provided by different OHS service agencies on the market are reasonably similar. Therefore, it is difficult for enterprises to judge the real quality of OHS services based on the price of those services alone.

### 3.2. Activity Simulation and Multiagent Model Building for the OHS Service Market

#### 3.2.1. Behavioral Decision-Making Design of Factory_Agent Purchasing OHS Services

The system creates* m* Factory_agents, each of which has to make the decision whether or not to purchase OHS services. The Factory_agents can choose to independently complete their OHS-related work. That is, they can adopt the no-service-purchasing strategy* j*_*1*_, or they can seek professional OHS services from professional OHS service agencies, which is effectively adopting the service-purchasing strategy* j*_*2*_. In the former case, the Factory_agents have to pay a certain safety cost for putting in place the OHSAS18001 safety system. In the latter case, they have saved the safety cost, but, due to the asymmetry of market information, they face the risk of failing to truly bring the OHSAS18001 safety system into play, due to the possibility of purchasing low-quality services. The revenue functions of a Factory_agent under the two strategies are* U*_*i*_*(j*_*j*_) (*j*=1,2) as, respectively, given below:

(1) Uij1=FFactory−agentst−CFactory−agentst

(2) Uij2=FFactory−agentst−Pst−βD

The study adopts the research of safety economy [45, 46], function *F*_*Factory*-*agent*_*(S) *represents safety income of enterprises, and function *C*_*Factory*-*agent*_* (S)* represents safety cost of enterprises. Safety income includes safety reduction* L(S)* and safety increase* I(S)*, *F*_*F*actory−*agent*_(*s*_*t*_) = *L*(*s*) + *I*(*s*). Safety reduction* L(S) *represents when safety level* s *increases, damage and loss will reduce; function is (3). Safety increase* I(S)* represents when safety level* s* increases, the service life of equipment extends and productivity increases; function is (4). Safety cost *C*_*Factory*-*agent*_* (S)* represents when safety level* S* increases, safety cost will be higher; function is (5).* L,l, I, i, L*_0_*, C*_*F*_, *C*_0_*F*__ are constant. If the purchased OHS services have failed to genuinely bring the OHSAS18001 safety system into play, the Factory_agent will face the risk of accidents. To be specific, when* β=1*, this means that an accident has occurred. When* β=0*, no accident has occurred. Also,* P(S)* represents the purchasing price of OHS services, and* D* represents the accident losses suffered by the Factory_agent.

(3) Ls=L·el/s+L0L>0,  l>0,  L0>0

(4) Is=I·e−i/sI>0,  i>0

(5) CFactory−agents=CF·ecF/1−s+C0FCF>0,  C0F>0

This paper adopts the EWA learning algorithm [47] to depict enterprise behaviors. The algorithm assumes that each strategy has a numerical attractiveness index, and introduces certain rules to determine the probability of selecting each strategy. See the specific updating formula below:

(6) NFt=ρNFt−1+1

(7) AFijjt=NFt−1φAFijjt−1+∂+1−∂IFjjUijjNFt

wherein *N*_*F*_(*t*) represents the empirical weight;* ρ *represents the discount factor of past experience; *A*_*F*_*i*__^*j*_*j*_^(*t*) represents the attractiveness index of the strategy *j*_*j*_  (*j=1,2*) to the Factory_agent; that is, the higher the value, the higher the probability of adopting this strategy; *φ* represents the discount factor of past attractiveness index; *U*_*i*_(*j*_*j*_) represents the expected revenue of the Factory_agent, where the Factory_agent will update the corresponding revenue according to its specific status; ∂ represents the discount factor of future strategy payments or opportunity costs; that is, the higher the value of ∂, the higher the importance attached to or the expectations held for the strategy by the Factory_agent. Finally, *I*_*F*_(*j*_*j*_) is an indicative function, where *I*_*F*_(*j*_*s*_) = 1 means that the strategy is adopted; thus

(8) $$\begin{array}{c} A_{F_{i}}^{j_{j}}\left(\;t\;\right)=\frac{N_{F}\left(t-1\right)\varphi A_{F_{i}}^{j_{j}}\left(t-1\right)+U_{i}\left(j_{j}\right)}{N_{F}\left(t\right)} \end{array}$$

When *I*_*F*_(*j*_*j*_) = 0, it means that the strategy is not adopted; thus

(9) $$\begin{array}{c} A_{F_{i}}^{j_{j}}\left(\;t\;\right)=\frac{N_{F}\left(t-1\right)\varphi A_{F_{i}}^{j_{j}}\left(t-1\right)+\partial U_{i}\left(j_{j}\right)}{N_{F}\left(t\right)} \end{array}$$

In the EWA learning algorithm, the attractiveness index will determine the probability of each strategy being selected. In other words, the higher the attractiveness index, the higher the probability of the strategy concerned being selected. This paper uses the logit response function [48] to express the probability of strategy *j*_*j*_ being selected by the Factory_agent, wherein *λ* is used to measure the sensitivity of the attractiveness index in decision-making. When* j=1*, it means that enterprise i has a probability of *P*rob_i_^*j*_1_^(*t* + 1) of choosing not to purchase service strategy *j*_1_ in phase t+1. When s=2, it means that enterprise i has a probability of *P*rob_i_^*j*_2_^(*t* + 1) of choosing to purchase service strategy *j*_2_ in phase* t+1*.

(10) $$\begin{array}{c} Prob_{i}^{j_{j}}\left(\;t+1\;\right)=\frac{exp\;\left(\lambda A_{F_{i}}^{j_{j}}\left(t\right)\right)}{\sum_{j=1}^{2}exp\;\left(\lambda A_{F_{i}}^{j_{j}}\left(t\right)\right)} \end{array}$$

#### 3.2.2. Occurrence of Factory_Agent Accidents

When low-quality OHS services are purchased, the OHSAS18001 safety system of the Factory_agent has failed to be genuinely brought into play. In such cases, the Factory_agent faces a certain occurrence probability of accidents. Given that the safety level* S*_*t*_ * (S*_*t*_*<1)* of the Factory_agent is inversely proportional to the occurrence probability of accidents, the model (for the purpose of better embodying the randomness of accidents) adopts a roulette mode to simulate the occurrence of accidents, as detailed below:

① Step 1: Determine the safety state of the Factory_agent according to its safety level* S*_*t*_, as indicated in Table 1.
② Step 2: Generate the random number R=random(0,1).
③ Step 3: Compare R with* S*_*t*_. *R* ≤ *S*_*t*_, there is no accident. When *S*_*t*_ ≤ *R* < 1, an accident occurs.

#### 3.2.3. Behavioral Decision-Making Design of Agency_Agent Providing OHS Services

Agency_agents' aim is to provide professional OHS services to Factory_agents and to thus help the latter realize and improve their OHSAS18001 safety systems and elevate their overall safety levels. However, the provision of OHS services by Agency_agents to Factory_agents is a market behavior. Agency_agents (as market agents assuming sole responsibility for their profits or losses) take the maximization of profits as their primary objective. At the beginning of the experiment, the system creates* n* Agency_agents, and the total profit made by each Agency_agent in each phase is determined by the profit from a single business transaction and the volume of business transactions. Theoretically, the size of the total profit is inversely proportional to the cost of a single business transaction and is directly proportional to both the profit from a single business transaction and the volume of business transactions. To be specific, the higher the quality of services provided by Agency_agents, the higher the business cost and, correspondingly, the lower the profit from a single business transaction. Thus, Agency_agents have the motivation to provide low-quality services. However, the size of the total profit is influenced not only by the profit from a single business transaction, but also by the volume of business transactions. Due to the asymmetry of market information, it is difficult for Agency_agents to judge how to make the optimal decisions that can attract more business and maximize their profits. As a result, Agency_agents will constantly adjust their behavioral decision-making in each cycle, based on market changes and previous experience. In the same manner, the EWA learning algorithm is employed to depict the behavioral decision-making of Agency_agents. To be specific, the decisions made by Agency_agents with regard to the provision of OHS services include three types, namely, (1) improving the quality of OHS services, (2) keeping the quality of OHS services constant, and (3) reducing the quality of OHS services. The expected revenue functions corresponding to the three types of decisions are *π*_*i*_(*k*_*j*_) (*j=1,2,3*), as, respectively, given below:

(11) πik1=Ts+·Qs+−θ·IV

(12) πik2=Ts·Qs−θ·IV

(13) πik3=Ts−·Qs−−θ·IV

wherein* T(S*^+^),* T(S)*, and* T(S*^−^), respectively, represent the volumes of current market businesses acquired in the three cases described above and* Q(S*^+^),* Q(S)*, and* Q(S*^−^), respectively, represent the profits from a single business transaction made in the three cases described above. When* θ=1*, this means that there is government intervention. When* θ=0*, there is no government intervention. In addition,* IV *represents the government intervention value. Assuming that $\overset{-}{s}$ represents the upper limit of service quality for government intervention, when $s<\overset{-}{s}$, Agency_agents are punished by the intervention value* IV*. When $s>\overset{-}{s}$, however, Agency_agents are supported by the intervention value* IV*. Assuming that the OHS services provided by Agency_agents in each case can elevate the safety level of Factory_agents to* s*, the profit from a single business transaction can be expressed as

(14) $$\begin{array}{c} Q\left(\;s\;\right)=P\left(\;s\;\right)-C_{Agency-agent}\left(\;s\;\right) \end{array}$$

Here,* P(s)* represents the price of services provided by Agency_agents and *C*_*Agency*-*agent*_*(s) *represents the business cost paid by Agency_agents [34] (normally, the lower the quality of services provided, the lower the business cost to be paid); that is, *C*_*Agency*−*agent*_(*s*) = *C*_*A*_ · *e*^[*c*_*A*_/(1−*s*)]^ + *C*_0__*A*_. The updated formula of the learning algorithm [35] adopted is given below:

(15) NAt=ρNAt−1+1

(16) AAikjt=NAt−1ϕAAikjt−1+∂+1−∂IAkjπikjNAt

wherein *N*_A_(*t*) represents the empirical weight; *A*_A_*i*__^*k*_*j*_^(*t*) represents the attractiveness index of strategy *k*_*j*_  (*j=1, 2, 3*) to the Agency_agent; *π*_*i*_(*k*_*j*_) represents the expected revenue of the Agency_agent; and *I*_A_(*k*_*j*_) is an indicative function, where *I*_A_(*k*_*j*_) = 1 means that the strategy is adopted; thus

(17) $$\begin{array}{c} A_{A_{i}}^{k_{j}}\left(\;t\;\right)=\frac{N_{A}\left(t-1\right)\phi A_{A_{i}}^{k_{j}}\left(t-1\right)+\pi_{i}\left(k_{j}\right)}{N_{A}\left(t\right)} \end{array}$$

When *I*_*A*_(*k*_*j*_) = 0, this means that the strategy is not adopted; thus

(18) $$\begin{array}{c} A_{A_{i}}^{k_{j}}\left(\;t\;\right)=\frac{N_{A}\left(t-1\right)\phi A_{A_{i}}^{k_{j}}\left(t-1\right)+\partial \pi_{i}\left(k_{j}\right)}{N_{A}\left(t\right)} \end{array}$$

Similarly, the logit response function is adopted to express the probability of the Agency_agent selecting strategy* K*_*j*_, wherein* λ* is used to measure the sensitivity of the attractiveness index in decision-making. When* j=1*, this means that service agency* i* has a probability of *P*rob_i_^*k*_1_^(*t* + 1) of choosing strategy* K*_*1*_ in phase* t+1*. That is, the quality of OHS services is improved. When* j=2*, this means that service agency* i *has a probability of *P*rob_i_^*k*_2_^(*t* + 1) of choosing strategy* K*_*2*_ in phase* t+1*. That is, the quality of OHS services remains constant. When* j=3*, this means that service agency* i *has a probability of *P*rob_i_^*k*_3_^(*t* + 1) of choosing strategy* K*_*3*_ in phase* t+1*. That is, the quality of OHS services is reduced.

(19) $$\begin{array}{c} Prob_{i}^{k_{j}}\left(\;t+1\;\right)=\frac{exp\;\left(\lambda A_{A_{i}}^{k_{j}}\left(t\right)\right)}{\sum_{j=1}^{3}exp\;\left(\lambda A_{A_{i}}^{k_{j}}\left(t\right)\right)} \end{array}$$

#### 3.2.4. “Death” and “Birth” of Agency_Agent

In the real world, a service agency may withdraw from the market for very complicated reasons. This model, for simulation purposes, has provided simplification to a very high degree. In our model, when the Agency_agent meets one of the following conditions, this means that the Agency_agent is forced to withdraw from the market.

① When the actual revenue of the Agency_agent is less than 0 for* T* consecutive phases (that is, when *π*_*i*_ ≤ 0), the Agency_agent withdraws from the market.

② According to the model, each Agency_agent has a fixed asset of* GT(t)*. In addition, the total asset* KT(t)* is the sum of the fixed asset combined with the profit of each phase, that is, *KT*(*t*) = *GT*(*t*) + ∑*π*. In cycle* t*, when the debt of the Agency_agent (i.e., penalty amount of punishment ∑*IV*_*punishment*_) exceeds the Agency_agent's current total asset* KT(t)* by a given proportion k (that is, when ∑*IV*_*punishment*_/*KT*(*t*) > *k*), the Agency_agent goes bankrupt for reasons of insolvency.

Corresponding to the death of a certain number of Agency_agents in any given cycle, new Agency_agents will enter the market. In our model, we assume that the entry of new Agency_agents depends on the average profit level of the entire industry. In each cycle, there are several randomly generated service agencies which have the required willingness to enter the market. Also, the higher the average profit level of the entire industry, the higher the probability of these service agencies entering the market.

#### 3.2.5. Behavioral Decision-Making Design of Government_Agent Intervention

As the “night watchman” of the market, the government must adopt the role of maintaining market order and preventing market failure. In the OHS service market of SMEs, the purchase of OHS services is a spontaneous behavior on the part of enterprises. The government has no right to interfere with the decisions of enterprises about whether or not those enterprises purchase OHS services, or which service agency they choose. However, unlike the case of an ordinary consumer market, in the OHS service market of SMEs, the effect of OHS services presents a severe lagging effect, as well as information asymmetry. Combined with the multiscale complexity of safety-related accidents, it is very difficult for SMEs to distinguish rights and liabilities via commercial contracts, as they could in the case of ordinary products. This combination of factors makes it even more difficult for SMEs to either distinguish service quality or investigate and affix the responsibility for the poor effect of OHS services. This creates an opportunity for service agencies to provide low-quality services. However, providing low-quality services not only causes unnecessary losses to enterprises, but also jeopardizes fair competition in the OHS service market. On that account, one governmental regulatory agency (Government_agent) is set in the system, and government intervention behaviors are introduced to explore the influence of government intervention on the quality of OHS services.

Based on related studies and real-life situations, this paper significantly simplifies and summarizes government intervention behaviors as three types, i.e., (1) government punishment, (2) policy supports, and (3) quality rating. The behavior of government punishment is manifested as follows: The Government_agent inspects the service quality of Agency_agents in the system and sets the upper limit (or standard* S*_*p*_) of service quality which must be met to avoid government punishment. That is, when the Government_agent finds that the quality of OHS services provided by some Agency_agents is lower than the standard* S*_*p*_, those Agency_agents are punished. The behavior of policy supports is manifested as follows: The Government_agent sets the lower limit* S*_*r*_ of service quality for policy supports and offers policy supports to Agency_agents in the system whose service quality is higher than* S*_*r*_. The behavior of quality rating is manifested as follows: The Government_agent regularly inspects the service quality of all Agency_agents and determines quality ratings on the Agency_agents on this basis. Factory_agents can observe the results of the quality ratings and thus more clearly understand the service quality of Agency_agents. Through the setting up of related parameter situations, we conducted experiments, respectively, to probe the influences of the different types of intervention behavior on OHS services.

### 3.3. Experimental Program

In this paper, Netlogo is used to simulate the action that Government-agent investigates the service quality of Agency-agent in the system based on the model constructed above. Three interventions including government punishment, policy support, and quality assessment are introduced to study the effect on OHS services of small- and medium-sized enterprises in Experiment I, Experiment II, and Experiment III, respectively.

In Experiment I, three sets of comparative experiments are further set up. To be specific, Experiment 1.1 is designed with two situations, namely, the situation before introducing government punishment and the situation after introducing government punishment. Then, the two situations are compared in terms of the average evolution of the overall quality of OHS services, as well as the change in the number of SMEs purchasing OHS services. Experiment 1.2 further verifies the influence of different punishment standards on OHS services after introducing government punishment. By setting the punishment standard* S*_*p*_, the evolutions of the quality of OHS services under the two situations are compared. Experimental 1.3 verifies the influence of different punishment intensities on OHS services after introducing government punishment. By setting the punishment intensity *β*, the evolutions of the quality of OHS services under the two situations are compared.

In Experiment II, three sets of comparative experiments are further set up too. Experiment 2.1 is designed with two situations, that is, the situation before introducing policy supports and the situation after introducing policy supports. Then, the two situations are compared in terms of the average evolution of the overall quality of OHS services and the change in the number of SMEs purchasing OHS services. Experiment 2.2 further verifies the influence of different support standards on OHS services after introducing policy supports. By setting the policy support standard* S*_*r*_, the evolutions of the quality of OHS services under the two situations are compared. Experimental 2.3 verifies the influence of different support intensities on OHS services after introducing policy supports. By setting the support intensity coefficient *α*, the evolutions of the quality of OHS services under the two situations are compared.

To observe the influence of the OHS services quality rating strategy introduced by the government, Experiment III is designed with two situations, namely, the situation before introducing quality rating and the situation after introducing quality rating. To be specific, in the situation after introducing quality rating, we assume that the Government-agent regularly inspects the service quality of Agency_agents and conducts quality ratings of all Agency_agents. Factory_agents can observe the results of these quality ratings and thus can obtain the evolution of service quality of Agency_agents by experiments.

## 4. Experiments and Results

### 4.1. Experiment I: Influence on the OHS Services of Small- and Medium-Sized Enterprises after Introducing Government Punishment

The first step is to create the experimental samples, that is, 1000 Factory_agents, 20 Agency_agents, and one Government_agent. The Agency_agents have an initial service level of* S*_*0*_ (wherein *S*_0_ ∈ (0,1)). Government_agent inspects the service quality of Agency_agents, and* S*_*p*_ is the upper limit of service quality adopted by the Government_agent for carrying out punishment, or, in other words, the punishment standard. When it is found that the quality of OHS services provided by some Agency_agents is lower than the standard* S*_*p*_, the low-service quality Agency_agents are punished. Also, *β* represents the punishment intensity coefficient. That is, the higher the coefficient, the higher the penalty amount. Setting Income Agency_agent as the current revenue of the Agency_agent, the punishment value* IV* punishment can be expressed as *IV*_*punishment*_ = *β* · *Income*_*Agency*_.

### 4.2. Experiment 1.1: Evolution of OHS Service Quality in Both the Situation with Government Punishment and the Situation without Government Punishment, and the Proportion of Enterprises Purchasing OHS Services

**Figure 2:**
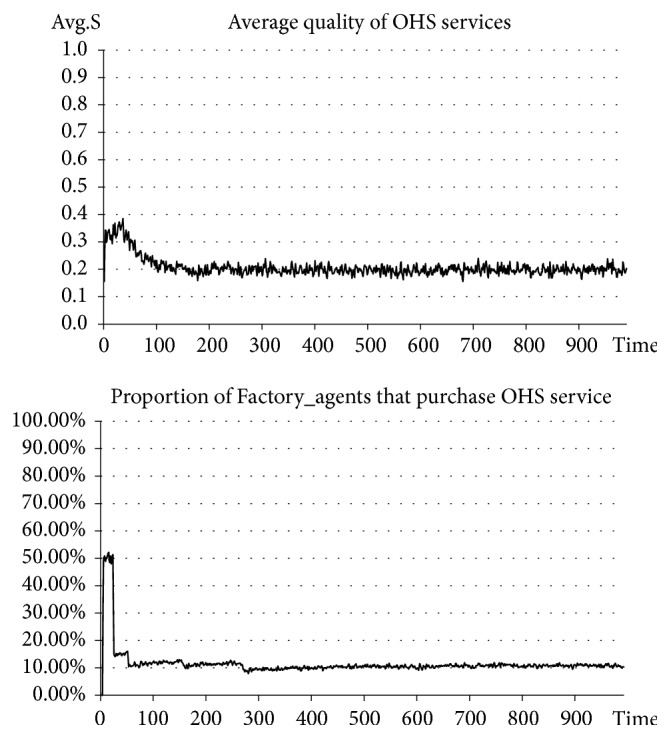
Situation before introducing government punishment.

**Figure 3:**
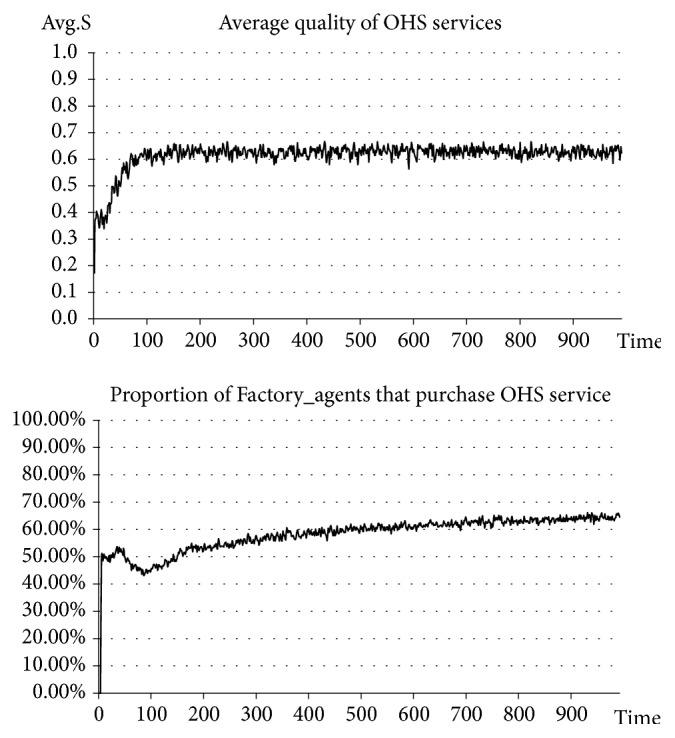
Situation after introducing government punishment.

In the situation before introducing government punishment, Government_agent-related activities are excluded. In the situation after introducing government punishment, Government_agent-related activities are added. The punishment intensity is set at the same time at* β=0.8*, and the upper limit of the quality of OHS services for government punishment, or the punishment standard, is set at* S*_*p*_*=0.6*. See the experimental results in Figures 2 and 3.

The results of our experiment indicate that, before introducing government punishment, the average quality of OHS services stabilizes at approximately 0.2, which is a relatively low level. In this case, the number of SMEs purchasing OHS services accounts for approximately 10% of the total number (a relatively small percentage). However, after introducing government punishment, the average quality of OHS services stabilizes at approximately 0.6-0.7, which is a higher level than before. In this case, the number of SMEs purchasing OHS services continuously rises and ultimately stabilizes at approximately 65% (an obviously and significantly increased percentage). Clearly, after introducing government punishment measures, the quality of OHS services has seen an apparent improvement and has also driven the rise of the number of SMEs purchasing OHS services. In other words, the improvement of the quality of OHS services can drive the growth of market demand for OHS services and promote the benign development of OHS services.

### 4.3. Experiment 1.2: Evolution of OHS Service Quality under a Certain Punishment Intensity but with Different Punishment Standards

**Figure 4:**
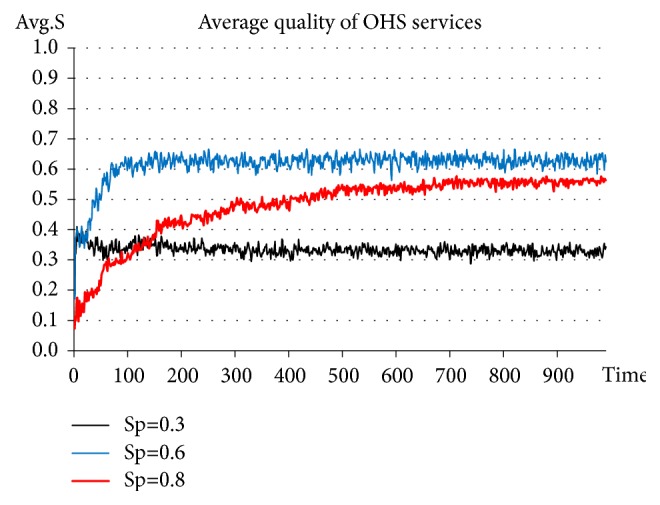
Evolution of OHS service quality in the situation of setting the punishment standard at 0.3, 0.6, and 0.8, respectively.

On the basis of adding Government_agent-related activities, the punishment intensity is set constantly at* β=0.8*. The upper limits of the quality of OHS services for government punishment (or the punishment standard) are set at* S*_*p*_*=0.3*, 0.6, and 0.8, respectively. See the experimental results in Figure 4.

The results of this experiment indicate that when the punishment standard is set at 0.3, the average quality of OHS services stabilizes at approximately 0.3-0.4. When the punishment standard is set at 0.6, the average quality of OHS services stabilizes at approximately 0.6-0.7. When the punishment standard is set at 0.8, the average quality of OHS services slowly rises up to approximately 0.5-0.6. Clearly, it is preferable to have the punishment standard set at a proper level, i.e., neither too low nor too high. When the punishment standard* S*_*p*_ set by the Government_agent is too high, it becomes difficult for most Agency_agents to significantly improve their service quality within a short period of time. In other words, when the government makes decisions regarding a punishment strategy, the punishment standard established should conform to the present situation pertaining to the strength of most service agencies. This will truly and effectively stimulate service agencies to improve the quality of their OHS services and to thus provide better OHS services for SMEs.

### 4.4. Experiment 1.3: Evolution of OHS Service Quality under a Certain Punishment Standard but with Different Punishment Intensities

**Figure 5:**
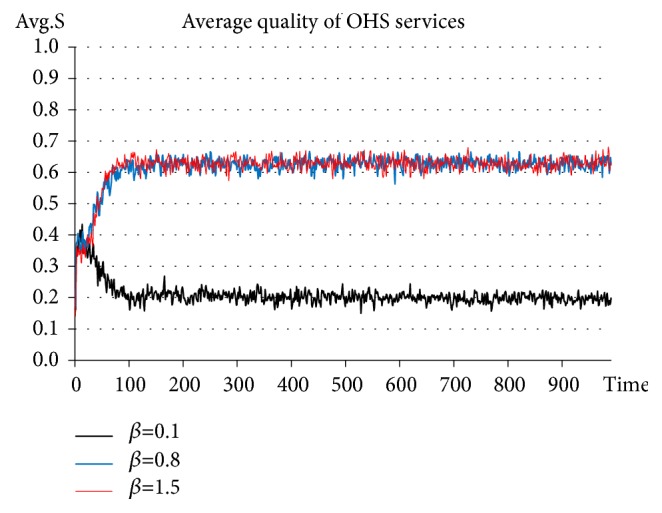
Evolution of OHS service quality in the situation of setting the punishment intensity coefficients at 0.1, 0.8, and 1.5.

On the basis of adding Government_agent-related activities, the government punishment standard is set constantly at* S*_*p*_*=0.6*. The punishment intensity coefficients are set at* β=0.1*, 0.8, 1.5, respectively. See the experimental results in Figure 5.

The results of this experiment indicate that when the punishment intensity is set at 0.1, the average quality of OHS services stabilizes at approximately 0.2. When the punishment intensity is set at 0.8, the average quality of OHS services stabilizes at approximately 0.6-0.7. When the punishment intensity is set at 1.5, the average quality of OHS services also stabilizes at approximately 0.6-0.7. Clearly, when the punishment intensity is set at 0.8, the level of OHS services quality is improved. In contrast, when the punishment intensity is set at the lower level (*β=0.1*), the quality of OHS services continuously trends downward, and government regulation fails to exert a positive influence. When the level of punishment intensity is much higher (*β=1.5*), the quality of OHS services is similar to that set at 0.8; however, a plenty of service agencies are found to die for excessively high pressure, with the experiment in progress. It is thus obvious that, when adopting a punishment strategy, the punishment intensity should be set at a proper level, so as to effectively promote the improvement of the quality of OHS services.

### 4.5. Experiment II: Influence on the OHS Services of Small- and Medium-Sized Enterprises after Introducing Policy Supports

The first step is to create the experimental samples, that is, 1000 Factory_agents, 20 Agency_agents, and one Government_agent. The Agency_agents have an initial service level of S_0_ (wherein *S*_0_ ∈ (0,1)). We assume that Government_agent offers supports to Agency_agents who are active in the market and whose service quality is above* S*_*r*_, wherein* S*_*r*_ represents the lower limit of service quality adopted by the Government_agent for carrying out support. The support value* IV*_*support*_ is expressed as *IV*_sup*port*_ = *α* · (*s*_*t*_ − *s*_*r*_) · *Income*/*m*, wherein *α* represents the support intensity coefficient,* “Income” *represents the total revenue of all service agencies, and* “Income/m”* represents the average revenue. That is, the higher the quality* S*_*t*_ of the OHS services provided by service agencies, the higher the value of policy supports those service agencies will receive.

### 4.6. Experiment 2.1: Evolution of OHS Service Quality in Both a Situation with Policy Supports and a Situation without Policy Supports, and the Proportion of Enterprises Purchasing OHS Services

**Figure 6:**
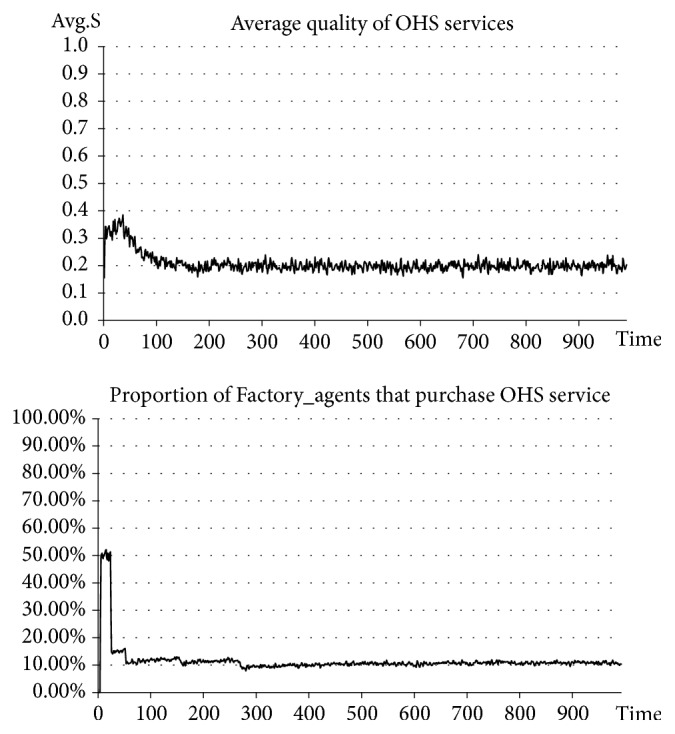
Situation before introducing policy supports.

**Figure 7:**
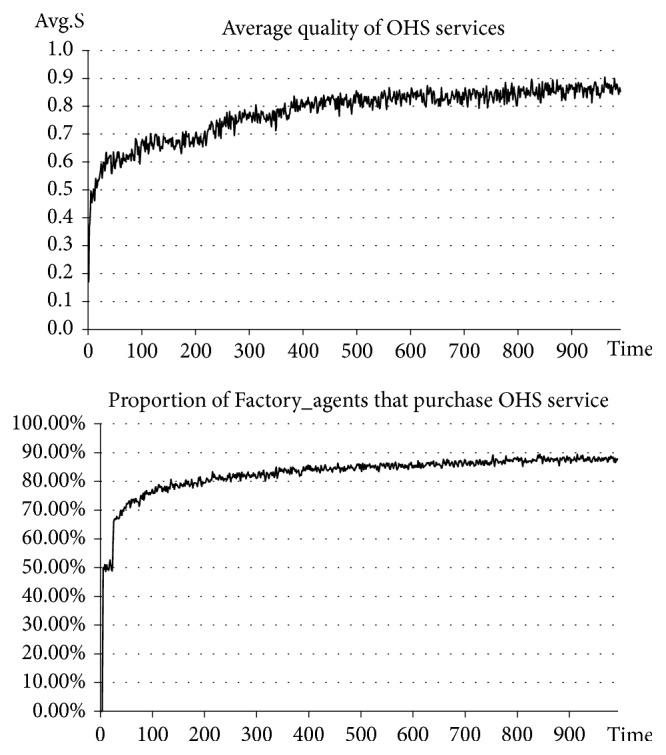
Situation after introducing policy supports.

In the situation before introducing policy supports, Government_agent-related activities are excluded. In the situation after introducing policy supports, Government_agent-related activities are added, and the support intensity coefficient is set at* α=1.5*. The lower limit of the quality of OHS services for policy supports from the government (or the support standard) is set at* S*_*r*_*=0.3*. See the experimental results in Figures 6 and 7.

The results of this experiment indicate that, before introducing policy supports, the average quality of OHS services stabilizes at approximately 0.2 (a relatively low level), in which case the number of SMEs purchasing OHS services accounts for approximately 10% of the total number (a relatively small percentage). However, after introducing policy supports, the average quality of OHS services stabilizes at approximately 0.8-0.9 (a relatively high level on the whole), in which case the number of SMEs purchasing OHS services is relatively large and stabilizes at approximately 89%. Clearly, after introducing policy support measures, the quality of OHS services has seen an apparent and significant improvement. This once again proves that the improvement of the quality of OHS services can effectively drive the growth of the market demand for OHS services and promote the positive development of OHS services.

### 4.7. Experiment 2.2: Evolution of OHS Service Quality under a Certain Support Intensity but with Different Support Standards

**Figure 8:**
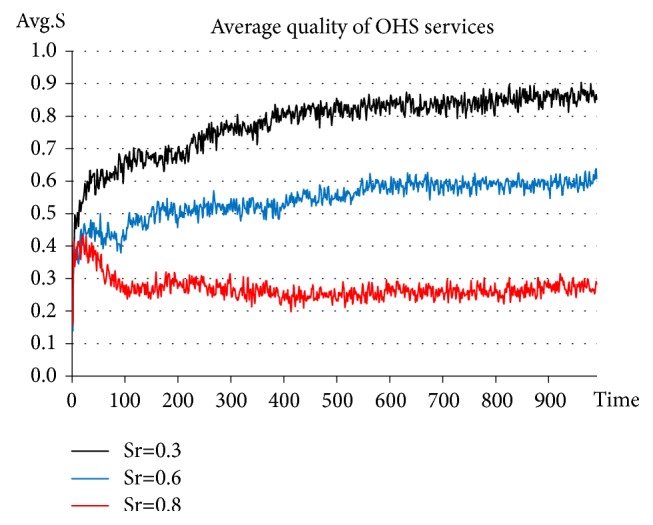
Evolution of OHS service quality in the situation of setting the support standards at 0.3, 0.6, and 0.8, respectively.

On the basis of adding Government_agent-related activities, the support intensity coefficient is set constantly at* α=1.5*. The policy support standards of the government are set at* S*_*r*_*=*0.3, 0.6, and 0.8, respectively. See the experimental results in Figure 8.

The results of this experiment indicate that when the support standard is set at 0.3, the average quality of OHS services rises up to approximately 0.8-0.9. When the support standard is set at 0.6, the average quality of OHS services stabilizes at approximately 0.6. When the support standard is set at 0.8, the average quality of OHS services is approximately 0.2-0.3. Clearly, when the support standard is set at too high a level (*S*_*r*_*=0.8*), the higher standard fails to exert any influence on the quality of OHS services. This is because, when the standard becomes too high, it becomes difficult for most service agencies to live up to the criteria. In this case, the support value* IV*_*support*_ fails to exert the desired positive effect on service agencies. In other words, the support policies become ineffective. These results are also consistent with observations of real-life situations, which suggests that an excessively high support standard does not exert any positive effect on the improvement of the quality of OHS services. When the support standard is set at a relatively low level (*S*_*r*_*=0.3, 0.6*), the quality of OHS services provided by service agencies (influenced by the function of the support value* IV*_*support*_) becomes directly proportional to the support value. In other words, the lower the support standard is, the stronger the stimulation will be to improve the quality of OHS services.

### 4.8. Experiment 2.3: Evolution of OHS Service Quality under a Certain Support Standard but with Different Support Coefficients

**Figure 9:**
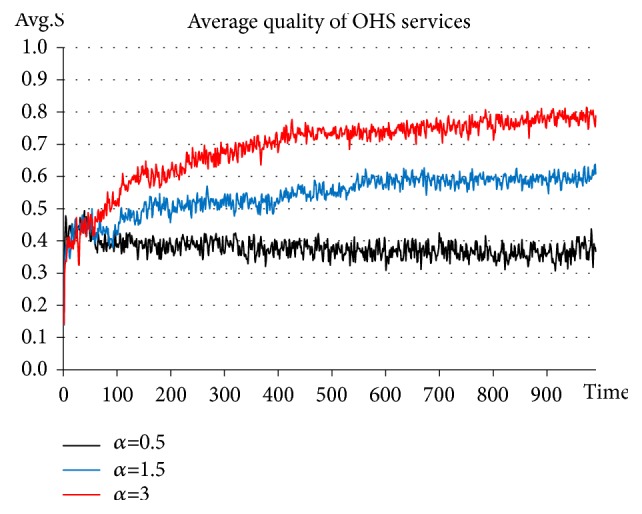
Evolution of OHS service quality in the situation of setting the support intensity at 0.5, 1.5, and 3.

On the basis of adding Government_agent-related activities, the support standard is set constantly at* S*_*r*_*=0.6*. The support intensity coefficients are set at* α=0.5, 1.5, 3*, respectively. See the experimental results in Figure 9.

The results of this experiment indicate that when the support intensity is set at 0.5, the average quality of OHS services stabilizes at approximately 0.3-0.4. When the support intensity is increased to 1.5, the average quality of OHS services increases as well and stabilizes at approximately 0.6. When the support intensity is increased to 3, the average quality of OHS services is continuously increased and stabilizes at approximately 0.8. As clearly shown by these experimental results, when the support intensity becomes too low, the quality of OHS services fails to achieve the expected effect, in which case policy supports become ineffective. Only when the support intensity has reached a certain level can policy supports be expected to exert their influence. In addition, the higher the support intensity, the stronger the stimulation effect on the improvement of the quality of OHS services. This is because excessively low support intensity results in an excessively low value of policy supports received by service agencies. The profit thereby generated is insufficient to offset the high cost of improving service quality. To put it in another way, the higher the support intensity, the higher the value of policy supports received by service agencies for improving the quality of their OHS services. When the support intensity reaches a certain level, the motivation of service agencies to improve the quality of their OHS services is significantly enhanced, thus promoting the all-round improvement of the quality of OHS services. These results are consistent with observations of real-life situations.

### 4.9. Experiment III: Influence on the OHS Services of Small- and Medium-Sized Enterprises after Introducing Quality Ratings

**Figure 10:**
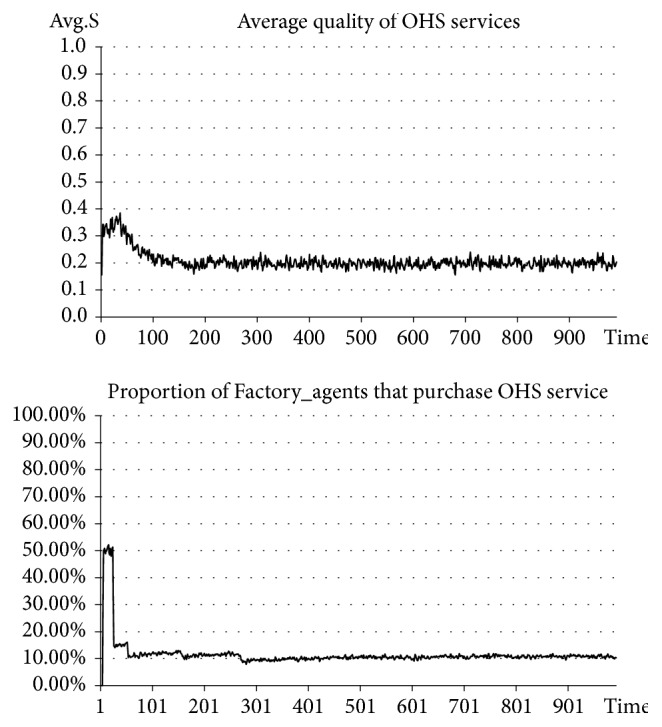
Situation before introducing quality rating.

**Figure 11:**
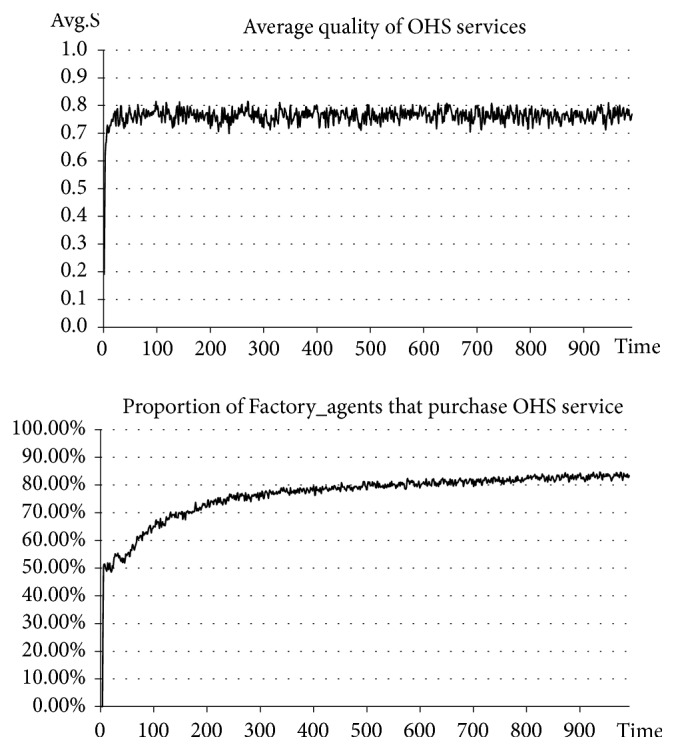
Situation after introducing quality rating.

The first step is to create the experimental samples, that is, 1000 Factory_agents, 20 Agency_agents, and one Government_agent. The Agency_agents have an initial service level of S_0_ (wherein *S*_0_ ∈ (0,1)). To observe the influence of the OHS services quality rating strategy introduced by the government, this experiment is designed with two situations, namely, the situation before introducing quality rating and the situation after introducing quality rating. To be specific, in the situation after introducing quality rating, we assume that Government_agent regularly inspects the service quality of Agency_agents and conducts quality ratings of all Agency_agents. Those whose service quality falls within the intervals of [0, 0.4], [0.4, 0.7], or [0.7, 1] are, respectively, rated at grade C, grade B, or grade A. Factory_agents can observe the results of these quality ratings and thus more clearly understand the service quality of Agency_agents. See the experimental results in Figures 10 and 11.

The results of our experiment indicate that, before introducing quality ratings, the average quality of OHS services stabilizes at approximately 0.2 (a relatively low level). In this case, the number of SMEs purchasing OHS services accounts for approximately 10% of the total number (a relatively small percentage). However, after introducing quality ratings, the average quality of OHS services stabilizes at approximately 0.8 (a relatively high level on the whole). In this case, the number of SMEs purchasing OHS services is obviously and significantly increased and stabilizes at approximately 80%. Clearly, when Factory_agents can observe the service quality of Agency_agents, they will actively seek cooperation with those who provide high-quality services. This in turn clearly shows that SMEs have a demand for high-quality OHS services. Introducing the quality rating strategy can effectively stimulate more SMEs to purchase high-quality OHS services and further promote the high-quality development of OHS services. This finding further proves that there is a mutually stimulating and interactive benign relationship between the quality of OHS services and the market demand for OHS services and that a positive developmental trend is hereby formed.

## 5. Discussions and Conclusions

This paper focuses on the intervention measures taken by the government in the development of the OHS market and demonstrates which intervention measures can achieve the real effective operating of the OHS service market. The effective operating of OHS service market means that enterprises are willing to buy services, and the services they bought are of high quality. The results of evolutionary experiments show that different interventions lead to different implementation effects and have different implementation conditions. Each intervention has certain constraints, advantages, and disadvantages. This means that the government should choose intervention measures according to actual circumstances. For example,

(1) Considering the effect of punishment strategy, the government should set appropriate punishment standard and intensity. It is difficult to stimulate the service agencies to improve service quality under excessive or too low punishment standard, or too low punishment intensity. Similarly, when the punishment intensity becomes too high, it is helpful to enhance the quality of service, but a large number of service agencies will die, which will be nonconducive to market development.

(2) Appropriate policy support standard and intensity of government are beneficial. Excessive support standard led to the fact that most service agencies are difficult to meet the requirements. In such cases, policy supports cannot truly exert any significant positive influence. Meanwhile, it is also not desirable to set the support intensity at too low a level, as the profit generated in this case is insufficient to offset the high cost of improving service quality. In other words, the expected effect of stimulating the improvement of service quality fails to be achieved. Therefore, according to the current level of market service quality, government should reduce support standards to improve support, so that service agencies are effectively supported by policy support, which promotes the service agencies to enhance the quality of OHS service.

(3) When the government introduces quality ratings of OHS services and discloses the results of such ratings, enterprises can actively seek cooperation with those who provide high-quality services by observing their service quality rating. This measure could also stimulate the significant improvement of the quality of OHS services and drive service market development.

Besides that, by comparison of the different governmental interventions, all the government interventions can effectively improve the quality of service under certain circumstances, simultaneously, increase the number of enterprises which buy OHS services, and ensure the good development of the market. However, each intervention has its advantages and disadvantages. From the perspective of the operation of the OHS service market, policy support strategy is the most effective way. It can motivate to a great extent the service agencies to improve their service quality and ensure the proper development of the market as long as the government reduces the support standard and increase the support intensity. In comparison to policy support strategy, the punishment strategy calls for extra attention to the standard and the intensity. The strategy would be ineffective if the standard and the intensity are set too low; however, when the standard and the intensity are set too high, the service agencies would have difficulty in greatly improving their services in a short period and would have to withdraw from the market at last, which goes against the prospect of sound operation of the OHS service market. From the prospective of the practicality of policy implementation, policy support strategy demands a great amount of fund from the government, which is rather difficult in reality. In contrast, punishment strategy and quality ratings strategy only incur costs in quality verification, and this is rather more feasible. By comparison, it can be seen that policy support strategy is the most effective way to promote OHS service development, however, which is at the cost of high regulatory expenses. Punishment strategy is similarly effective, but it will bring too much pressure to the OHS service agencies. Quality ratings strategy is not only effective to the OHS service agencies, but also economic to the government.

Our study, however, is still fallible to limitations. First of all, we have set a general situation to achieve the simulation. On the basis of reality, we assume that the cost of SMEs' self-built OHS system is higher than that of purchasing OHS services; SMEs are unable to tell the quality of OHS services from the price; and quality OHS services are bound to improve the corporates' OHS quality. Therefore, the results of this study are valid in general situations, and the results may deviate in special situations when the postulated conditions change. Secondly, our study only takes governmental intervention strategies into consideration and does not introduce other interested agents into the playing field, which, though it goes beyond this study, is very important for future studies. Thirdly, in regard to the setting of quality rating strategy, this essay only verifies whether the strategy yields quality rating effects. In the future, the author will delve into the way of information disclosure of different rating systems.

Despite all these limitations, this research develops a rigid experimental framework dedicated to analyzing the influence of different intervention strategies on SMEs' OHS service quality. It also provides an evolution experiment, with three specific strategies applicable to various situations, to examine the influence of different governmental intervention strategies on SMEs' OHS service quality. Every strategy experiment consists of one or several control experiments. The results of the experiments can tell the positive influence that government interventions exert on OHS service quality and how to control the standard and the intensity of various strategies to achieve the optimum results.

In consideration of the experiment results, this paper argues that, in order to ensure the operation of OHS service market so as to help SMEs build the OHSAS18001 system efficiently, the government should take intervention strategies to control OHS service quality and the strategies may be reasonable and moderate, as the Chinese SMEs' OHS management is lagging far behind the OHSAS18001 standards. Meanwhile, the intervention should fit the Chinese national conditions and the current situation of the service market, for instance, in the initial stage of the market, if the government has a good finance, is willing to invest, and adopts incentive methods that will activate the energy of the market. When the market researches a certain scale, the government can adopt the strategies of punishment and quality rate information exposure, strengthen the service quality supervision of service organizations, encourage service organizations to improve service quality, standardize behavior of the market, and guide good operation of the market. Therefore, considering environment of the market and the supervision cost, the government can choose the most appropriate intervention, prevent the failure of OHS service market, stimulate development of the market, and finally maintain the OHS level of SMEs.

## Figures and Tables

**Table 1:** Safety state table of Factory_agent.

State	Safe	Unsafe
Probability	S_t_	1-S_t_
Cumulative probability	S_t_	1

## Data Availability

The data used to support the findings of this study are available from the corresponding author upon request.
